# Face-to-face vs. remote digital settings in job assessment interviews: A multilevel hyperscanning protocol for the investigation of interpersonal attunement

**DOI:** 10.1371/journal.pone.0263668

**Published:** 2022-02-07

**Authors:** Michela Balconi, Giulia Fronda, Federico Cassioli, Davide Crivelli

**Affiliations:** 1 International Research Center for Cognitive Applied Neuroscience (IrcCAN), Università Cattolica del Sacro Cuore, Milano, Italy; 2 Research Unit in Affective and Social Neuroscience, Department of Psychology, Università Cattolica del Sacro Cuore, Milano, Italy; Universiti Tunku Abdul Rahman, MALAYSIA

## Abstract

The digitalization process for organizations, which was inevitably accelerated by the COVID-19 pandemic, raises relevant challenges for Human Resource Management (HRM) because every technological implementation has a certain impact on human beings. Between many organizational HRM practices, recruitment and assessment interviews represent a significant moment where a social interaction provides the context for evaluating candidates’ skills. It is therefore relevant to investigate how different interaction frames and relational conditions affect such task, with a specific focus on the differences between face-to-face (FTF) and remote computer-mediated (RCM) interaction settings. In particular, the possibility of qualifying and quantifying the mechanisms shaping the efficiency of interaction in the recruiter-candidate dyad—i.e. interpersonal attunement—is potentially insightful. We here present a neuroscientific protocol aimed at elucidating the impact of FTF vs. RCM modalities on social dynamics within assessment interviews. Specifically, the hyperscanning approach, understood as the concurrent recording and integrated analysis of behavioural-physiological responses of interacting agents, will be used to evaluate recruiter-candidate dyads while they are involved in either FTF or RCM conditions. Specifically, the protocol has been designed to collect self-report, oculometric, autonomic (electrodermal activity, heart rate, heart rate variability), and neurophysiological (electroencephalography) metrics from both inter-agents to explore the perceived quality of the interaction, automatic visual-attentional patterns of inter-agents, as well as their cognitive workload and emotional engagement. The proposed protocol will provide a theoretical evidence-based framework to assess possible differences between FTF vs. RMC settings in complex social interactions, with a specific focus on job interviews.

## Introduction

In recent years, the interest of neuroscience for social, cognitive, and affective phenomena has turned to the organizational-managerial domain. Among others, constructs such as leadership [1–4], decision-making processes [5,6], and communication [7,8] were highly investigated. Due to the limitations imposed by the COVID-19 pandemic (e.g., stay home restrictions), in some jurisdictions, companies were required to adopt alternative communication methods to perform most of their organizational processes. Among many changes in *human resource management* (HRM), a sector where interpersonal relationship is pivotal, the selection process, if not suspended, was mostly carried out remotely. Selecting and evaluating a candidate for a company placement holds a significant value for business success and represents a driver in the long term for added value. It is also economically essential because it can limit cases of bad hiring and loss of good candidates [9]. Through the various phases of the selection process, the interview represents an important position as a direct social interaction touch point, which also allows the evaluation of the candidate’s social skills, a good predictor of employability [10].

We believe that this technological and organizational change happened rapidly and could also be seen as an opportunity to gather insights on how human interaction is modulated by digital communication systems, as well as on the effect of computer-mediated interaction contexts on social dynamics and their psychological, cognitive, and neurophysiological correlates.

Moreover, attention to neuroscientific applications for human-centred technology has grown, with an interest in *human-computer interaction* (HCI) [11] and how the interpersonal relationship might be impacted by technology [12]. The increased use of digital communication tools, the pervasiveness of virtual environments for interaction, and the sudden turn towards new spaces for social engagement have an active role in shaping the relationship between people [13]. As an example, given the intrinsic limitations in properly conveying part of non-verbal communication cues shared by some of the most diffused web-based communication and conferencing platforms (degraded information on body posture, proxemics and gestures if a webcam is used, loss of information apart from verbal and vocal ones if no webcam is used), the massive use of such digital tools has gradually modified the information exchange, inter-personal regulation, and sense-making processes that connote HRM duties, thus leading to a greater focus on linguistic content and, among non-verbal communication channels, on non-verbal vocal cues (e.g. prosody), paralinguistic contents, eye-behaviour, and facial expressions.

It is then questionable how computer-mediated interactions provide the right physical, social, and psychological setting and to what extent they are adequate for building engagement, which represents the foundation of interpersonal relations and modulates their development over time. Available empirical findings are controversial. According to Waytz & Gray [14], technology for communication could work both as a social connector and separator. In fact, while online communication minimizes the social cues characterizing face-to-face exchanges, that might also positively affect some forms of interpersonal interaction [e.g. 15] and might help people for whom face-to-face interaction is perceived as challenging and stressful [16]. Also, remote communication could help increase accessibility by, as an example, removing potential barriers due to individual resources for transportation, other forms of personal limitations, or social inequalities. At the same time, it was also reported that online communication might be associated with decreased empathy [17] and increased individualism [18]. And again, many individual factors—such as age, generation, and technology use habits—also seem to contribute to the subjective effects of such mediated kinds of interaction [14]. Indeed, it is necessary to understand how interactions mediated by digital devices negatively influence interpersonal interaction by stressing the inter-agents. In particular, recently, the interest regarding the possible positive and negative effects of the interaction between individuals and digital technologies has increased, with a specific focus on the prevention and reduction of digital distress—i.e. technostress—which can have adverse consequences on psychological, physical, and physiological levels [19,20]. The prevention of technostress is essential, especially in this historical period in which, following the Covid-19 pandemic, the use of digitally-enhanced technologies and processes has exponentially entered many social situations and interaction opportunities both in everyday life and the workplace, including personnel selection and recruiting.

Focusing on the use of digital communication technologies in recruiting, screening, and selection for job candidates, previous studies [e.g. 21] have already pointed out how such technologies might affect those critical processes. However, to the authors’ best knowledge, the systematic investigation of neurofunctional correlates of face-to-face and computer-mediated interactions and, in particular, of possible differences between such communication modalities when used in professional contexts for selection and recruitment purposes is as yet unexplored, notwithstanding its potential to develop and promote informed practices.

Regarding those two modalities, we believe that it is relevant to study the mechanisms underlying the complex interaction that develops in the recruiter-candidate dyad, so as to carry out an in-depth analysis of its evolution across its core steps (i.e. introduction to the company and presentation of the candidate; aptitude test and colloquium; test and assessment of the candidate’s technical skills and expertise; conclusion and feedback on the assessment interview) and of its effectiveness by qualifying and quantifying explicit and implicit markers of interpersonal attunement.

In this light, the protocol has been designed to investigate oculometric (gaze plot, scan path), autonomic (electrodermal activity, EDA; heart rate, HR; heart rate variability, HRV), and neurophysiological (electroencephalography, EEG) correlates of interpersonal and inter-brain synchronization in recruiter-candidate dyads involved in assessment interviews carried out in face-to-face (FTF) *vs*. remote computer-mediated (RCM) interaction settings. Indeed, gaze behaviour is considered a core marker of the quality of interpersonal dynamics since gaze is a primary way to influence and re-direct the attention focus of the inter-agent, to regulate social roles and hierarchy, to both seek and provide feedback from/to the inter-agent in order to adjust the interaction, and to aptly manage conversational turn-taking [22]. Concurrent investigation of eye behaviour in interacting agents might, therefore, provide highly valuable information on the efficiency of the information exchange and on the progress of the interaction. At the same time, autonomic markers of physiological arousal—namely, increased EDA, accelerated and less variable HR—indicate the modulation of the stress response as well as the cognitive-affective load imposed by a social interaction [23], especially when it is peculiarly sensitive as in the case of assessment interviews. EEG (i.e. non-invasive recording of electrophysiological correlates of neural activity via electrodes placed on the scalp) completes the picture by providing fine-grained data on central information-processing and emotional engagement via, specifically, the analysis of task-related synchronization/desynchronization in the theta, alpha, and beta frequency bands [24]. The remarkable temporal resolution of the EEG technique allows for accurate monitoring of the evolution of the dynamics of interpersonal exchange as well as the attunement between inter-agents. In particular, interpersonal attunement will be assessed by using the hyperscanning approach, an experimental methodology that allows, based on simultaneous recording of behavioural and physiological responses from different agents involved in a joint task or a social exchange, the computation of inter-agents synchronization and inter-brain coupling metrics mirroring the level of social attunement [25,26]. The hyperscanning technique has already proved to be a reliable and valuable way to explore the efficiency and quality of interpersonal relations and complex social exchanges, both in laboratory and ecological settings such as workplace [26–33].

Going down to specifics, we expect to observe: (i) greater focus of gaze behaviour on the face and the eyes of the inter-agent in the RCM than in the FTF condition, though not merely as a consequence of limited framing but rather as a marker of the search for relevant non-verbal cues to regulate the interaction; (ii) greater autonomic arousal activity in candidates who will be interviewed in the RCM setting with respect to FTF interviewing, mirroring greater affective distress due to the complexity imposed by the mediated context to the social attunement process; (iii) gradually decreasing autonomic activation in the RCM condition across the different steps of the assessment interview, mirroring attenuation and habituation effects; (iv) greater interpersonal synchronization and social attunement in FTF than in RCM relational contexts, as measured via inter-brain coupling metrics (EEG hyperscanning); (v) gradually increasing interpersonal attunement in RCM interaction across the different steps of the assessment interview, mirroring a slower though progressive synchronization process and the higher cognitive burden imposed by the mediated social contexts at least during the initial adjustment phase of the interaction.

## Materials and methods

This experiment is designed as a between-group, single-centre, experimental study. After enrolment, the research timesheet includes a 4-week data collection phase and, subsequently, a 6-week data processing and analysis phase.

The study and its experimental procedures follow the operational and ethical principles of the Declaration of Helsinki and its subsequent revisions. They were reviewed and approved by the Ethics Committee of the Department of Psychology of the Catholic University of the Sacred Heart (ref. 022021, approved on February 2021). Written informed consent will be obtained from all subjects. Participation in the study will be voluntary; enrolment will be managed independently of the companies that will provide the organizational context for the experimental tasks.

### Target population and sample

The population we aim to study is constituted by HR professional with well-established expertise in personnel selection and recruitment and by potential candidates for a job interview. HR professionals will be enrolled via snowball sampling given their peculiar profile that might make probabilistic sampling difficult. Potential candidates will be enrolled via voluntary response sampling. We aim at recruiting 34 recruiter-candidate dyads (see the *Statistical considerations* paragraph).

#### Inclusion criteria

Participants must meet three inclusion criteria, as follows:

i) over 18 years of age; ii) normal or corrected-to-normal vision and hearing; and iii) proficiency in using online communication technologies. Furthermore, HR professionals must meet the following additional criteria for inclusion: i) being currently employed in public/private companies; ii) being regularly involved in personnel selection and recruitment duties; and iii) have at least five years of professional experience in HR, personnel selection or recruitment.

#### Exclusion criteria

Participants exhibiting any of the following criteria will be excluded: i) having a history of neurology or psychiatry disorders; ii) being involved in concomitant therapies with psychoactive drugs that might affect the central nervous functioning; iii) presenting clinically relevant distress or history of burnout.

### Experimental procedure

Self-report, oculometric, autonomic, and neurophysiological metrics will be used to evaluate the quality of interaction in the dyads. For self-report metrics, psychometric tools will be used to assess personality and anxiety-related traits (see the Materials paragraph) while the outcome and quality of the interview between recruiter and potential candidate will be assessed via an experience sampling questionnaire. Also, the quality of interviews will be evaluated via the analysis of video recordings of the social exchanges, with a peculiar focus on the occurrence of non-verbal cues of psychophysical activation and conversational gestures (i.e. gestures that accompany speech and support it). Oculometric measures will be used to investigate both the inter-agents’ visual exploration patterns and potential differences in the weight of non-verbal cues in different interaction settings. As for autonomic and neurophysiological metrics, peripheral and central measures will both be included in hyperscanning data collection so as to gather multi-level information on the modulation of cognitive workload, emotional engagement, and arousal during the interaction, both within each inter-agent and in the dyad.

The semi-structured interview phase will last approximately 30 minutes and will follow a pre-defined outline including, specifically: a) an introductory phase dedicated to the presentation of the candidate and the company; b) an attitudinal phase based on vocational and aptitude tests and/or colloquium; c) an assessment phase dedicated to testing and discussion of the candidate’s technical skills and the requirements of the job position; and d) conclusions and feedback from the recruiter. Namely, recruiters will have a checklist of topics to be explored (the ones cited in the above-described interview phases), but the questions and communication flow will be freely managed during the interaction so as to try and foster engagement between the interlocutors and mutual connection. For the same reasons, no specific time limitation for different interview phases will be imposed. At the same time, the request of covering a fixed checklist of topics in the interview phases will allow for gathering comparable data across different interviews. Ocular, autonomic, and neurophysiological data will be concurrently collected from the two inter-agents constituting the interaction dyad during the whole duration of the interview, as well as at rest before the beginning and after the interview (2-minute resting-state baseline recording). Demographic, vocational, and psychometric data concerning personality traits, anxiety signs and technological proficiency will be collected one week before the interview takes place to minimize the risk for potential biases during the interview recordings. Finally, the experience sampling questionnaire concerning perceived communication efficacy, the quality of the interaction, perceived comfort, and engagement experienced during the interview will be administered at the end of the interaction.

The dyads will be randomly assigned to two experimental conditions. One group will carry out the assessment interview face-to-face (FTF condition), while the other one will carry out the interview via a digital platform for remote meetings (Microsoft Teams) using personal computers (RCM condition). Regardless of the randomly-assigned experimental condition, resting-state and interaction-related autonomic and neurophysiological measures will be collected in hyperscanning. Namely, inter-agents’ peripheral and central physiological responses will be concurrently recorded and then aligned offline so as to investigate their coherence and temporal correlation. To allow for such experimental paradigm to take place in properly controlled conditions, in the RCM condition we will ask both the recruiter and the potential candidate to come to the university campus, where they will be located in separate standard meeting rooms, preventing any physical contact between them and any knowledge of them being in the same location. In the FTF condition, both participants will instead be located in the same meeting room at the university campus. Given the novelty and explorative nature of the study and the consequent lack of previous evidence or established methods to refer to, we have opted for such forms of environment standardization in order to increase comparability between experimental conditions and to minimize potential confounds in data collection.

### Materials

#### Psychometric measures

In order to gather information on basic personality and anxiety-related traits of participants, as well as their technological proficiency, the initial screening procedure includes the short form of the revised Eysenk Personality Questionnaire [EPQ-r short form; 34], the Social Interaction Anxiety Scale [35], and a questionnaire on technological proficiency to explore participants’ familiarity, knowledge and expertise in using digital media, online resources, and software for web-based communication and remote conferencing/meeting platforms.

#### Experience-sampling questionnaire

Perceived communication efficacy, quality of the interaction, perceived comfort, and engagement experienced during the assessment interview will be explored via an experience sampling questionnaire at the end of the interaction. Namely, the inter-agents will be asked to rate, according to a 10-point Likert scale where 1 represents “Not at all” and 10 represents “Totally”, the overall pleasantness of the experience, how much they have found themselves at ease during the interview, the efficacy of communication during the interaction, the degree of engagement in the relationship with the other inter-agent, how much they have felt effective during the interaction, the degree of attunement with the other inter-agent, and the extent to which he/she thinks that the interaction condition has influenced the development of the interview.

#### Nonverbal coding of video recordings

Video recordings of the interviews were used to identify non-verbal cues of stress and psychophysical activation—i.e. adaptors, gestures and movements implemented, mainly out of conscious control, to improve comfort, reduce stress and/or manage nervousness—and of conversational flow—i.e. conversational gestures, mostly unaware acts and movements that co-occur with speech and support it by, as an example, stressing some passages [36]. Each video recording will be reviewed by two experts in non-verbal communication, who will keep note of the occurrence of such non-verbal cues and code them based on the category (adaptor vs. conversational gesture) and on the interview phase. All codes will be mutually exclusive in order to prevent ambiguous classifications. In case of discrepancies or ambiguous cases, the coders will discuss such instances during coding meetings with the research team leaders (the authors). Ambiguous instances will not be further considered in subsequent analysis if the coders could not reach a consensus. Discrepancies will be resolved by a third coder (one of the authors) so the dataset will reflect the consensus of at least two out of three coders. The dataset will include the occurrence of adaptors and conversational gestures during the four phases of the interview.

#### Eye-tracking and oculometric data

Data concerning eye-behaviour and gaze focus in the interacting dyads will be monitored and collected via non-invasive eye-tracking systems that, by using infrared-light and by measuring light reflection rates concerning the pupil and the cornea, creates a reconstruction of eye positions and movements during visual exploration. The eye-tracking systems will be calibrated on each participant, thus improving the accuracy of data collection.

Oculometric data will be processed offline via a dedicated software to derive the inter-agents’ attention patterns. Areas of interest (AoI, i.e. the areas of a visible scene that are of interest to the research team) will be created for each dyad, with a specific focus on inter-agents’ faces and eyes. Fixation count, fixation length, and time to first fixation data will be finally extracted for each of the four core steps of the assessment interview plot.

#### Autonomic recordings and processing

Autonomic activity will be concurrently recorded from both the recruiters and the candidates constituting the interaction dyads via multipurpose peripheral sensors, as required by the hyperscanning method. HR and HRV will be specifically collected via photopletismography. The recording sensor will be placed in correspondence to the distal phalanx of the second finger of the non-dominant hand. Data will be sampled at 40 Hz. Recordings will be preceded by an accommodation period to allow sensors to set on baseline levels and participants to habituate to the setting.

Collected signals will be screened offline for the presence of artefacts. Following artefact rejection and, if needed, data filtering to reduce noise, inter-beat interval (IBI) data will be computed starting from raw HR data so as to compute time-domain HRV metrics [23, sdNN; 37]. Phasic skin conductance responses (SCR) will also be extracted from tonic EDA activity via moving average [23,38]. Finally, mean HR, sdNN, mean EDA, and SCR count will be computed for both resting and for each of the four core steps of the assessment interview plot.

#### EEG recording and reduction

Recruiters’ and candidates’ EEG activity will be recorded, according to the hyperscanning methodology, via two EEG systems, by using a lean 15-channel montage. Electrodes will be placed in correspondence to F7, F3, Fz, F4, F8, T7, C3, Cz, C4, T8, P3, Pz, P4, O1, and O2 electrode sites [SI10;^39^], using Ag/AgCl sensors with physical reference to the earlobes. vEOG will also be monitored to keep track of eye blinks for subsequent signal processing. EEG data will be sampled at 1000 Hz, with a 0.01–200 Hz bandpass input filter and a 50 Hz notch filter. Electrodes’ impedance will be kept below 5 kΩ.

During offline processing, a 0.5–50 Hz bandpass filter will be applied to recorded raw data. Average reference will also be computed in order to reduce the potential effect of situational biases on recorded data and improve the comparability of inter-agents’ EEG. A regression-based ocular correction algorithm will be used to lower the impact of ocular movements and blinks on EEG tracks. Data will then be segmented according to the internal structure of the assessment interview (four steps) and screened for residual ocular and movement artefacts. Finally, power spectra will be computed via FFT transform (resolution 0.5 Hz) so as to extract power density data for the standard EEG bands: delta (0.5–3.5 Hz), theta (4–7.5 Hz), alpha (8–13 Hz), beta (13.5–30 Hz), and gamma (30–50 Hz). The average EEG power profile will be computed and extracted for both resting and for each of the four core steps of the assessment interview plot.

### Safety

Experimental procedures will require the acquisition of oculometric, autonomic, and electrophysiological indices via non-invasive recording techniques, as well as of behavioural responses. Electromedical devices that will be used in the study are certified for safety and application with human subjects and will be set and managed by expert researchers with specific training. Experimental sessions will be video recorded to allow for the monitoring of interaction dynamics and, in addition, in order to be able—in case of need following, for example, a loss of markers timing data—to recover timing and other useful information for the integrated analyses of the inter-agents’ behavioural and physiological responses. Data will be anonymised, ensuring the protection of participants. No primary risks are associated with participation.

### Statistical considerations

#### Sample size estimation

Since both the investigated phenomena (i.e., inter-personal attunement and synchronization of electrophysiological and autonomic activities in dyads of professional recruiters and potential candidates involved in face-to-face and remote assessment interviews) and the methodological approach (hyperscanning methods and synchronization measures) are still new to social neuroscience research and since literature still lacks of systematically reproduced evidences, we cannot refer to previous data to determine the size of expectable significant effects. Still, we know from previous pilot studies and previous experimental observations from hyperscanning studies based on EEG and autonomic measures that the effect of experimental manipulations of interaction dynamics via framing (e.g., manipulating reinforcing feedbacks on inter-agents performance during collaborative *vs*. competitive tasks) on central indices of inter-personal synchronization and on individual behavioural and physiological measures can be quite high [η2 > 0.3; e.g., 40]. We have then estimated the adequate sample size to detect large effects via inferential statistics (d = 1.0), with α error probability set at 0.05 and with 0.80 power [G*Power 3.1 software– 41,42]. The analysis suggested that a total of 34 observations—i.e., in our case, dyads—would be sufficient. We aim at stopping enrolment once we have obtained the estimated sample size, while keeping a waiting list of additional 5 dyads in case of dropouts or technical issues during data collection.

#### Statistical analysis

Three sets of analyses will be performed on experience-sampling, nonverbal coding of video recordings (occurrence of adaptors and conversational gestures), gaze behaviour (AoI fixation count, fixation length, time to first fixation), autonomic (EDA, HR, HRV), and neurophysiological (EEG power) measures.

In the first set of analyses, independent samples t-tests (main factor: Condition, FTF *vs*. RCM) will be applied to the item scores of the experience-sampling questionnaire filled by each candidate or recruiter participant, while nonverbal coding, oculometric, autonomic, and neurophysiological measures by each candidate or recruiter participant will, instead, be analysed via linear mixed-effects models including both Condition (two groups: FTF *vs*. RCM) and Step (four within-subject levels: Introduction, Attitudinal, Technical, and Conclusion) as fixed effects. In addition, the linear mixed-effects models devised to analyse EEG power data will also include Localization (four within-subject levels: frontal, central, temporal, vs. parietal area) and Lateralization (two within-subject levels: left vs. right) as fixed factors. Subjects will be included as random effect so as to control for potential confounding effects due to inter-individual differences. Autocorrelations between subsequent steps of the assessment interview will be modelled using a first-order autoregressive covariance matrix, so as to accurately run a trend analysis through the assessment interview steps. Conditional F-tests will be used to determine the significance of terms in fixed effects and simple-effects for significant interactions will be tested by pair-wise comparisons with Bonferroni correction for multiple comparisons. The Benjamini-Hochberg procedure [43] will also be used to check the outcomes of data analysis against the false discovery rate. The size of statistically significant effects will be estimated via *d* values following Cohen’s norms [44].

In the second set of analyses, proper inter-personal synchronization metrics for autonomic (EDA, HR, HRV) and neurophysiological (EEG power) data in each dyad will be quantified by computing the partial correlation coefficient Π_ij_ [a metric of functional connectivity, 45] starting from single-brain autonomic and EEG data collected during the interviews and normalizing the inverse of the covariance matrix Γ = Σ^−1^:

$$\Pi_{ij}=-\frac{\Gamma_{ij}}{\sqrt{\Gamma_{ii}\Gamma_{jj}}}$$

Potential condition-related or step-related differences in inter-personal synchronization metrics will then be analysed via linear mixed-effects models including subjects as random effect and Condition (two groups: FTF *vs*. RCM) and Step (four within-subject levels: Introduction, Attitudinal, Technical, and Conclusion) as fixed effects. Models applied to EEG data will also include Localization (four within-subject levels: frontal, central, temporal, and parietal area) and Lateralization (two within-subject levels: left vs. right) as additional fixed effects. A first-order autoregressive covariance matrix will be used to model autocorrelations between subsequent steps of the assessment interview. Again, conditional F-tests will be used to determine the significance of terms in fixed effects and simple-effects for significant interactions will be tested by pair-wise comparisons with Bonferroni correction for multiple comparisons. Even in this case, the false discovery rate will be controlled via the Benjamini-Hochberg procedure [43] and the size of statistically significant effects will be estimated via *d* values following Cohen’s norms [44].

In the third set of analyses, correlation coefficients (namely, Pearson bivariate correlation coefficients) will be computed on experience-sampling, nonverbal coding, and inter-personal synchronization data (both autonomic and neurophysiological ones) to explore the strength and direction of the association between these different explicit and implicit measures of quality of the interaction. Potential interpretation biases due to multiple comparisons will be accounted for by controlling the false discovery rate via the Benjamini-Hochberg procedure [43].

## Conclusions

The proposed protocol will provide a theoretical evidence-based framework to assess possible differences between face-to-face vs. remote settings in complex social interactions, with a specific focus on job interviews. With the specific metrics and data analysis process that we have described (concerning neuropsychological, neurophysiological, psychological and behavioural constructs), it will be possible to evaluate the quality of the interaction, the degree of interpersonal attunement and synchronization, and the efficacy of the interaction.

Should the experimental protocol prove to be sound, feasible, and informative, it could represent a first proof of concept for a novel multi-level assessment method to investigate complex real-life social exchanges even in more ecologically-valid contexts (e.g. field, workplace or home-based recordings). It could also provide a methodological reference for studying the impact on inter-personal attunement and synchronization of different degrees of social cues deprivation in remote digital interactions (e.g. audio only conversations, avatar-based interactions, and fully live interactions), which would in turn provide valuable information for the development of adaptive interactive interfaces and affective computing. Also, those working in HRM could benefit from the protocol’s insights into the strategy to adopt when managing the selection of candidates. This could be calibrated based on the business target while taking into account the strength and limitations of the interaction context.
